# Efficient generation of relativistic near-single-cycle mid-infrared pulses in plasmas

**DOI:** 10.1038/s41377-020-0282-3

**Published:** 2020-03-20

**Authors:** Xing-Long Zhu, Su-Ming Weng, Min Chen, Zheng-Ming Sheng, Jie Zhang

**Affiliations:** 1 Key Laboratory for Laser Plasmas (MOE), School of Physics and Astronomy, Shanghai Jiao Tong University, 200240 Shanghai, China; 2 Collaborative Innovation Center of IFSA, Shanghai Jiao Tong University, 200240 Shanghai, China; 3 SUPA, Department of Physics, University of Strathclyde, Glasgow, G4 0NG UK; 4 Cockcroft Institute, Sci-Tech Daresbury, Cheshire, WA4 4AD UK; 5 Tsung-Dao Lee Institute, 200240 Shanghai, China; 6 Institute of Physics, Chinese Academy of Sciences, 100190 Beijing, China

**Keywords:** Laser-produced plasmas, Nonlinear optics

## Abstract

Ultrashort intense optical pulses in the mid-infrared (mid-IR) region are very important for broad applications ranging from super-resolution spectroscopy to attosecond X-ray pulse generation and particle acceleration. However, currently, it is still difficult to produce few-cycle mid-IR pulses of relativistic intensities using standard optical techniques. Here, we propose and numerically demonstrate a novel scheme to produce these mid-IR pulses based on laser-driven plasma optical modulation. In this scheme, a plasma wake is first excited by an intense drive laser pulse in an underdense plasma, and a signal laser pulse initially at the same wavelength (1 micron) as that of the drive laser is subsequently injected into the plasma wake. The signal pulse is converted to a relativistic multi-millijoule near-single-cycle mid-IR pulse with a central wavelength of ~5 microns via frequency-downshifting, where the energy conversion efficiency is as high as approximately 30% when the drive and signal laser pulses are both at a few tens of millijoules at the beginning. Our scheme can be realized with terawatt-class kHz laser systems, which may bring new opportunities in high-field physics and ultrafast science.

## Lasers: Towards intense ultra-short infrared pulses

A method to produce ultra-short and intense pulses of mid-infrared laser light could open new opportunities in applications ranging from super-resolution spectroscopy to attosecond X-ray pulse generation and particle acceleration. The procedure is proposed and numerically demonstrated by researchers in China and the UK led by Su-Ming Weng and Zheng-Ming Sheng at Shanghai Jiao Tong University in China. The method is based on the interaction of two pulses of laser light with a plasma – a gaseous mix of ions and electrons. The researchers also describe procedures to flexibly tune the resulting mid-infrared pulses by adjusting the plasma and lasers. If the current simulations are confirmed in practical tests, the method could overcome difficulties found in using standard optical techniques to generate high intensity mid-infrared pulses of close to single-cycle duration.

## Introduction

Since the laser was invented in 1960^1^, it has become a powerful and important tool for various applications in fundamental science, industry, medicine, and so on. In particular, the invention of the chirped pulse amplification technique^2^ by Strickland and Mourou in 1985 dramatically boosted the intensity of laser pulses, usually at near-infrared (near-IR) wavelengths, to an unprecedented level. This revolutionary invention brought laser-matter interactions into the relativistic regime for the first time. It has produced the highest accelerating field and highest pressure on earth, which are comparable to those of energetic events in the universe, providing unprecedented opportunities for various scientific studies^3–8^. Because of their ultrashort durations (down to a near-single optical cycle^9,10^), these laser pulses also allow one to explore and control ultrafast processes in the microcosm^11^ and open the door towards attosecond science^12^ and nonlinear optics of a vacuum^9,10^. At present, however, relativistic ultrashort laser pulses are usually obtained in the near-IR range. There is increasing interest in extending these laser pulses to other wavebands, such as the mid-IR range. Ultrashort intense mid-IR pulses are particularly useful for ultrafast and high-field physics, chemistry, biology, and materials science, such as ultrahigh harmonic generation^13^, attosecond pulse radiation^14^, infrared spectroscopy^15,16^, high-resolution imaging of ultrafast molecular dynamics^17^, and filamentation^18^. When these mid-IR pulses are further enhanced to relativistic intensities, there will be many new opportunities for applications in particle acceleration^19–21^, high-field physics^22–24^, and the generation of brighter hard X-rays and shorter attosecond pulses^13,14,25^, all of which would greatly benefit from the long carrier wavelength, high peak intensity, few-cycle duration, and multi-millijoule (mJ) pulse energy. Therefore, diverse methods have been proposed for creating intense few-cycle mid-IR pulses by using nonlinear crystals^26–30^. However, they are currently limited to non-relativistic intensities. In particular, the generation of high-energy intense mid-IR pulses with a near-single cycle is highly challenging.

In recent years, plasma-based optical techniques have received broad interest. Since plasma-based optical elements can sustain much higher laser intensities than conventional crystal-based elements, they are extensively proposed for the generation and manipulation of high-intensity laser pulses. Novel concepts such as plasma mirrors^31^, plasma gratings^32^, plasma optical modulators^33^, plasma undulators^34^, and plasma optical polarizers^35^ have been proposed and/or demonstrated. In particular, the generation of intense mid-IR pulses via the self-modulation of ultrahigh-power relativistic laser pulses in plasmas has been verified numerically or experimentally by several groups^36–39^. However, they normally relied on Joule-class 100-terawatt (TW)-level laser facilities, which are still expensive and run at only a few-Hz repetition rate. Moreover, the resulting mid-IR pulse has a typical ultrabroad continuous spectrum, and the energy conversion efficiency is limited to a few percent or even less. These severely restrict their availability for wide practical applications. Therefore, it is of great importance to generate intense few-cycle mid-IR pulses with controllable spectra and at high efficiencies using compact high-repetition-rate laser systems, which could provide more stability and wider accessibility for a broad community.

To address this intriguing quest, we propose a scheme for the efficient generation of relativistic multi-mJ near-single-cycle mid-IR pulses based on a novel type of plasma optical modulator. It utilizes two co-propagating laser pulses in an underdense plasma, where one pulse drives a plasma wake as the frequency modulator and the other is incident with a certain time delay as the signal pulse to be frequency-downshifted. When the drive laser pulse is intense enough, the plasma wake is highly nonlinear and appears as a few bubbles behind the drive laser^40–42^. These moving plasma bubbles serve as ideal optical structures suitable for frequency modulation. As long as the signal pulse is properly loaded at the front of the second bubble, it can be converted to a mid-IR pulse at an obviously longer central wavelength of ~5 μm with a surprisingly high energy conversion efficiency of ~30%. More importantly, the generated mid-IR pulse can reach a relativistic intensity with an ultrashort duration of a near-single cycle.

## Results

### Concept for mid-IR pulse generation

**Fig. 1:**
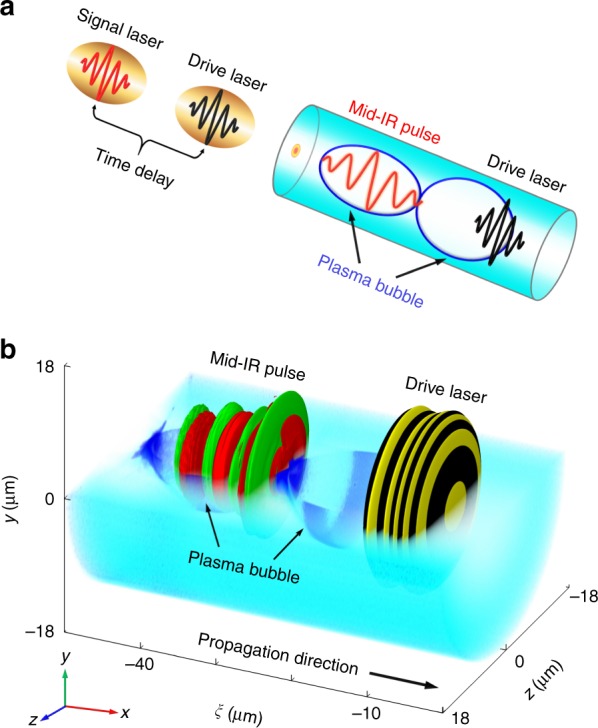
Concept of a plasma optical modulator for mid-IR pulse generation. This concept involves two laser pulses in an underdense plasma (see Methods section for the details of the initial parameters), where a drive laser pulse first excites a nonlinear plasma wake while a co-propagating signal pulse is injected into the second bubble of the wake. After a sufficient modulation time, the signal pulse is dramatically frequency-downshifted and converted into a mid-IR pulse, as seen in (**a** schematic diagram) and (**b** 3D simulation result), where *ξ* = *x* − *ct* is the variable in the light frame

Figure 1 shows a schematic of the relativistic few-cycle mid-IR pulse generation from a laser-driven plasma wake. First, an intense drive pulse propagates in an underdense plasma and creates a nonlinear plasma wake, which is composed of a few plasma bubbles moving at a phase velocity close to the group velocity of the laser pulse. Subsequently, a signal laser pulse initially with the same wavelength of the drive laser is incident into the plasma wake. The signal pulse is appropriately delayed to ensure that it loads at the front of the second plasma bubble. As the signal laser co-propagates with the plasma bubble, it undergoes a frequency downshift to a central wavelength extended to the mid-IR spectral range. Moreover, the frequency-downshifted pulse can propagate steadily in the plasma channel over many Rayleigh lengths (over 1.6 mm in our cases) along a stable pointing direction during the frequency-downshift process. Thus, this scheme is stable for the frequency downconversion of the input laser pulse in the plasma wake. More importantly, the drive pulse and the signal pulse in this scheme require only a few tens of mJ of energy and a few TW of peak power (see Methods section), which can be readily delivered by the existing compact multi-TW kHz-level laser systems^43–46^.

### Plasma optical modulation mechanism

The mechanism of mid-IR pulse generation via plasma-based optical frequency modulation can be described as follows. A plasma wave created by an intense laser pulse in an underdense plasma via a nonlinear wake-field excitation gives the change in the electron density *n*_e_(*ξ*, *τ*) and the plasma frequency *ω*_p_(*ξ*, *τ*) in time and space^47–49^. This leads to the change in the local phase velocity of light as $v_{\mathrm{p}}\left( {\xi ,\tau } \right) \approx c + c\omega _{\mathrm{p}}^2\left( {\xi ,\tau } \right)/2\omega ^2$ according to the dispersion relation $\omega ^2\left( {\xi ,\tau } \right) = c^2k^2\left( {\xi ,\tau } \right) + \omega _{\mathrm{p}}^2\left( {\xi ,\tau } \right)$ for the local instantaneous laser frequency *ω*(*ξ*, *τ*), where *ξ* = *x* − *ct*, *τ* = *t*, and *c* is the speed of light in a vacuum. When the laser pulse resides in the density up-ramp region of the plasma wave, the local phase velocity increases along the laser propagation direction since d*v*_p_/d*ξ* ∝ ∂[*n*_e_(*ξ*, *τ*)]/∂*ξ* > 0, where ∂[*n*_e_(*ξ*, *τ*)]/∂*ξ* is the gradient of the electron density perturbation, which gives rise to the change in frequency or wavelength of the laser pulse.

Theoretically, the local variation in the wavelength within a short period of time *dτ* can be estimated by^50^ ${\mathrm{d}}\lambda = {\Delta }v_{\mathrm{p}}{\mathrm{d}}\tau$, where ${\Delta}v_{\mathrm{p}} \approx \lambda \partial v_{\mathrm{p}}/\partial \xi$ is the phase velocity difference between two adjacent light wave crests (or troughs) and $\frac{{\partial v_{\mathrm{p}}}}{{\partial \xi }} \approx \frac{c}{{2n_{\mathrm{c}}}}\left( {\frac{\lambda }{{\lambda _0}}} \right)^2\frac{{\partial n_{\mathrm{e}}\left( {\xi ,\tau } \right)}}{{\partial \xi }}$; thus, one has

1 $${ \frac{{{\mathrm{d}}\lambda}}{{{\mathrm{d}}\tau}} \approx \frac{{c\lambda }}{2n_{\mathrm{c}}}\left( {\frac{\lambda }{{\lambda _0}}} \right)^2\,\frac{{\partial n_{\mathrm{e}}\left( {\xi ,\tau } \right)}}{{\partial \xi }} }$$

where $n_{\mathrm{c}} = m_{\mathrm{e}}\omega _0^2/4\pi e^2$ is the critical plasma density, *ω*_0_ = 2*πc*/*λ*_0_ is the initial laser frequency, and *e* and *m*_*e*_ are the electron charge and mass, respectively. This equation can be written in the form of $\lambda ^{ - 3}{\mathrm{d}}\lambda \approx \frac{c}{{2n_{\mathrm{c}}\lambda _0^2}}\frac{{\partial n_{\mathrm{e}}\left( {\xi ,\tau } \right)}}{{\partial \xi }}{\mathrm{d}}\tau$. The integral of this equation gives $\frac{1}{{\lambda _0^2}} - \frac{1}{{\lambda ^2}} \approx \frac{c}{{n_{\mathrm{c}}\lambda _0^2}}\int_0^T \frac{{\partial n_{\mathrm{e}}\left( {\xi ,\tau } \right)}}{{\partial \xi }}{\mathrm{d}}\tau$, where *T* is the interaction time. As a consequence, the wavelength of the laser pulse modulated by the plasma wave can be estimated as

2 $${ {\lambda} \approx \lambda _0\left( {1 - \frac{c}{{n_{\mathrm{c}}}}\int_0^T \frac{{\partial n_{\mathrm{e}}}}{{\partial \xi }}{\mathrm{d}}\tau } \right)^{ - 1/2} }$$

When $\frac{c}{{n_{\mathrm{c}}}}\int_0^T \frac{{\partial n_{\mathrm{e}}}}{{\partial \xi }}{\mathrm{d}}\tau \ll 1$, it gives ${\lambda} \approx \lambda _0( {1 + \frac{c}{{2n_{\mathrm{c}}}}\int_0^T \frac{{\partial n_{\mathrm{e}}}}{{\partial \xi }}{\mathrm{d}}\tau })$. Equation (2) suggests that the signal laser pulse will be frequency redshifted when it resides in a region of increasing density, thus potentially producing a mid-IR pulse in the wake. This suggestion is demonstrated by our simulations, as detailed below.

It is noted that the difference in the phase velocity of a probe light wave in regions of different plasma density has already been maturely used in experiments to measure the electron density perturbation of a plasma wake in the so-called pump-probe interferometry^51–53^, which is a promising tool for the on-line monitoring and control of plasma-based accelerators. This suggests that our scheme can be realized experimentally with the existing optical technology.

### Relativistic few-cycle mid-IR generation

**Fig. 2:**
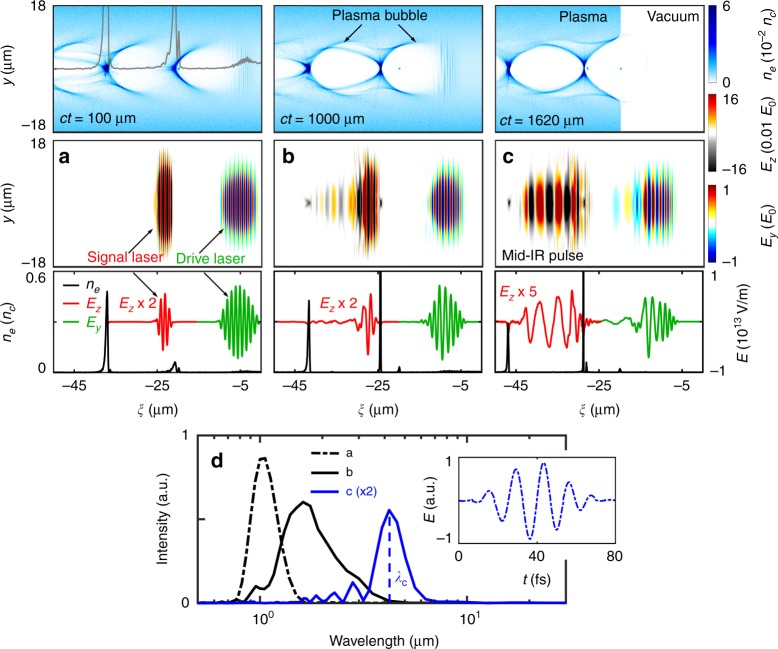
Illustration of the relativistic few-cycle mid-IR generation mechanism. The plasma electron density (*n*_e_) in the first row and the transverse electric fields of the drive laser (*E*_y_) and the signal laser (*E*_z_) in the second row are shown at different positions *ct* = 100 μm (**a**), 1000 μm (**b**) and 1620 μm (**c**). Their corresponding on-axis distributions are shown in the third row. **a** The gray line in the density map shows the on-axis density profile. The transverse electric fields and the density are normalized to *E*_0_=*m*_e_*cω*_0_/*e* and *n*_c_, respectively. **d** The spectrum evolution of the modulated signal pulse, corresponding to the positions in (**a** black dashed line), (**b** black solid line) and (**c** blue solid line). The inset shows the temporal waveform of the output mid-IR pulse at the central wavelength *λ*_c_ ≈ 4.2 μm. The laser field for the signal pulse has been multiplied by a factor of 2, 2, and 5 (red lines) in **a**–**c**, respectively, and the spectral intensity, by a factor of 2 (blue line) in **d**

We demonstrate this concept using fully three-dimensional (3D) relativistic particle-in-cell (PIC) simulations (see Methods section). Figure 2a–c illustrates the evolution of the drive pulse, the signal pulse and the plasma wake in the plasma-based optical modulation. The relativistic drive laser first creates a nonlinear wake as it propagates in the plasma; then, the signal laser enters the wake and resides in the density up-ramp of the second bubble with ∂[*n*_e_(*ξ*, *τ*)]/∂*ξ* > 0 (Fig. 2a). Due to the ponderomotive force of the signal laser pulse, the electron sheath density at the front of the second bubble further increases and results in a sharp density gradient, as shown in Fig. 2b. As a result, the signal pulse experiences a strong wavelength elongation or frequency-downshifting to a spectral peak at ~1.7 μm (Fig. 2d). After a sufficient modulation time, the infrared pulse is further frequency-downshifted to a spectral peak at *λ*_c_ ≈ 4.2 μm. The produced infrared photons (with a low frequency *ω*_ir_ = 2*πc*/*λ*_ir_ < *ω*_0_) quickly slip backwards to the center of the second bubble because of their relatively slower group velocity $v_{\mathrm{g}} = c( {1 - \omega _{\mathrm{p}}^2/\omega _{{\mathrm{ir}}}^2})^{1/2}$. As soon as the resulting mid-IR pulse arrives at the bubble center, it is trapped there, and its spectrum undergoes little change, as shown in Fig. 2c. Therefore, this plasma optical modulator is ideal for creating and maintaining intense long-wavelength infrared photons. In this example, the resulting pulse with a 4.2 μm central wavelength has a two-cycle full width at half maximum (FWHM) short pulse duration and a normalized amplitude $a_{{\mathrm{ir}}} = \frac{{\lambda _{\mathrm{ir}}E_{{\mathrm{z,ir}}}e}}{{2\pi m_{\mathrm{e}}c^2}} \approx 1.3$, which is well above the relativistic intensity threshold. The final signal pulse at the 4.2 μm central wavelength in the spectral range of 3–6 μm retains approximately 30% of the initial signal pulse energy after the frequency downshift, which is surprisingly high. More interestingly, its energy loss mainly contributes to the enhancement of the plasma wake when it resides in a density up-ramp region in the second bubble, which in return promotes the frequency-downshifting. Compared to the strong frequency-downshift of the signal pulse, only a small part of the rear edge of the driver laser pulse undergoes a moderate photon frequency-downshift with a conversion efficiency of ~1% in a spectral range above 3 μm (see Fig. S1 in the Supplementary Information for the spectral evolution of the driver pulse). This occurs because only a small part of the drive laser pulse is located in the density up-ramp region at the front of the first bubble, and here, the density gradient is very gentle. Furthermore, the whole plasma wake excitation is caused mostly by the drive laser, which depletes its substantial energy.

**Fig. 3:**
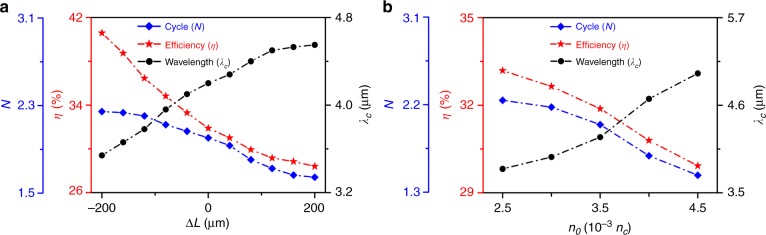
Effects of the plasma parameters on the output mid-IR pulse. The mid-IR optical cycle number (*N*), energy conversion efficiency (*η*) and central wavelength (*λ*_c_) evolution with changing **a** channel length *L* = Δ*L* + *L*_0_ (with *L*_0 _= 1597 μm and *n*_0_ = 3.5 × 10^−3^*n*_c_) and **b** plasma density *n*_0_ (with fixed *n*_0_*L* = 5.59 μm · *n*_c_)

We investigated the effects of the plasma length and density on mid-IR pulse generation. Figure 3a presents the dependence of the number of optical cycles, energy conversion efficiency and central wavelength of the produced infrared pulse on the plasma channel length $L = {\mathrm{\Delta }}L + L_0$, where Δ*L* is the length change relative to *L*_0_ = 1597 μm. Other plasma and laser parameters are the same as those in Fig. 2 unless otherwise stated. It is shown that the infrared wavelength increases nearly linearly with the plasma length as *λ*_ir_ ∝ *L* when *L* is relatively short. With a further increase in *L*, however, the central wavelength becomes saturated at *λ*_c_ ~ 4.5 μm. This saturation occurs because the plasma density defines the size of the plasma bubble $\left( {\lambda _{\mathrm{b}} \approx 2\pi \sqrt {a_0} /k_{\mathrm{p}}} \right)$, which puts an upper limit on the wavelength of the trapped infrared photons. In addition, an overlong plasma will lead to a significant attenuation and energy depletion of the pulse.

In Fig. 3b, we consider the effect of the plasma density on the infrared pulse generation, where the product of the plasma length and density is fixed with $n_0L = 5.59\,\mu {\mathrm{m}} \cdot n_{\mathrm{c}}$ to prevent excessive laser absorption in the plasma and to sustain an appropriately long interaction distance for photon frequency downconversion. In this case, the wavelength change may scale as d*λ* ∝ ∂[*n*_e_(*ξ*, *τ*)]/∂*ξ* according to Eq. (1). Since a sharper density gradient is excited in a denser plasma, it gives rise to faster frequency-downshifting with a significant wavelength elongation. This is verified by our simulations; for example, when using a higher plasma density *n*_0_ = 4.5 × 10^−3^*n*_c_, the infrared central wavelength is increased to *λ*_c_ ~ 5 μm, with a duration of a near-single optical cycle. However, it is worth noting that the plasma density should not be too high; otherwise, the plasma bubble shrinks dramatically as *λ*_b_ ∝ $\sqrt {a_0/n_{\mathrm{e}}}$ such that it does not have sufficient volume to accommodate the produced long-wavelength photons. A high-density plasma will also lead to a significant pulse attenuation due to strong absorption. In addition, the generated low-frequency infrared photons are subject to the plasma cutoff frequency constraint; hence, they may not be able to propagate in a high-density plasma.

**Fig. 4:**
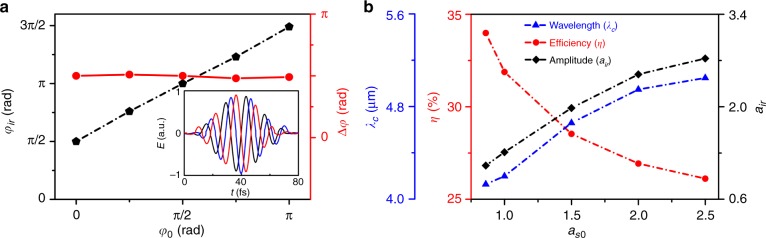
Dependence of the output mid-IR pulse parameters on the intensity and CEP of the initial signal laser. **a** Dependence of the CEP of the output infrared pulse (*φ*_ir_) on that of the initial signal pulse (*φ*_0_) for the case presented in Fig. 2. The inset shows the electric field waveform of the *λ*_c_ ≈ 4.2 μm infrared pulse for different CEPs of the initial signal pulse (0, black line; *π*/2, blue line; *π*, red line). **b** The infrared pulse central wavelength (*λ*_c_), normalized amplitude (*a*_ir_), and energy conversion efficiency (*η*) as functions of the signal laser intensity

We have also investigated the robustness of this scheme in terms of the carrier-envelope phase (CEP), intensity and spot size of the input signal pulse. Figure 4a shows the evolution of the CEP of the infrared pulse as a function of the CEP of the initial signal laser. As an ultrashort laser pulse propagates in a plasma of density perturbation, such as in a plasma wave with time-dependent density *n*_e_(*τ*) and $\omega _{\mathrm{p}}\left( \tau \right) = \sqrt {4\pi e^2n_{\mathrm{e}}(\tau )/m_{\mathrm{e}}}$, a shift in the pulse CEP will occur^50^. This shift is attributed to the plasma dispersion or, more precisely, the difference between the phase velocity (controlling the carrier phase) $v_{\mathrm{p}} \approx c[1 + \omega _{\mathrm{p}}^2\left( \tau \right)/2\omega ^2] \,>\, c$ and the group velocity (controlling the envelope) $v_{\mathrm{g}} \approx c[1 - \omega _{\mathrm{p}}^2\left( \tau \right)/2\omega ^2] \,<\, c$. The shift in the carrier phase of the pulse defined at the peak point of the laser electric field after a propagation time *T* can be estimated by ${\mathrm{\Delta }}\varphi \approx {\int}_0^T {(v_{\mathrm{p}} - v_{\mathrm{g}})} \omega /cd\tau$. Therefore, the shift in the carrier phase of the mid-IR pulse (*φ*_ir_) relative to that of the signal pulse (*φ*_s_) in the plasma wave is given by $\varphi _{{\mathrm{ir}}} - \varphi _s \approx {\int}_0^T {\left[ {(v_{\mathrm{p}} - v_{\mathrm{g}})\omega _{{\mathrm{ir}}}(\tau )/c - (v_{\mathrm{p}} - v_{\mathrm{g}})\omega _0/c} \right]{\mathrm{d}}\tau }$, where *ω*_ir_(*τ*) is the central instantaneous frequency of the infrared (modulated) pulse. Since $\varphi _{\mathrm{s}} - \varphi _0 \approx {\int}_0^T {(v_{\mathrm{p}} - v_{\mathrm{g}})} \omega _0/c{\mathrm{d}}\tau$, one has the resulting change in the carrier phase between the output infrared pulse and the initial signal pulse:

3 $${{\mathrm{\Delta }}\varphi = \varphi _{{\mathrm{ir}}} - \varphi _0 \approx {\int}_0^T {(v_{\mathrm{p}} - v_{\mathrm{g}})} \omega _{{\mathrm{ir}}}(\tau )/c{\mathrm{d}}\tau}$$

where *φ*_0_ is the initial carrier phase of the signal pulse. Further, by inserting $\omega _0^2 = 4\pi e^2n_{\mathrm{c}}/m_{\mathrm{e}}$, *v*_p_ and *v*_g_ into Eq. (3), one can obtain ${\mathrm{\Delta }}\varphi \approx {\int}_0^T {\frac{{n_{\mathrm{e}}(\tau )}}{{n_{\mathrm{c}}}}} \frac{{\omega _0^2}}{{\omega _{{\mathrm{ir}}}(\tau )}}{\mathrm{d}}\tau$. For a given driver laser and a fixed delay between the driver laser and the signal laser, both *n*_e_(*τ*) and *ω*_ir_(*τ*) are determined, which are independent of the initial carrier phase *φ*_0_ of the signal pulse. As a result, Δ*φ* is independent of *φ*_0_. This outcome agrees with our simulation results given in Fig. 4a, where ${\mathrm{\Delta }}\varphi _{{\mathrm{ir}}} \approx 0.5\pi$ under the given conditions, which is independent of *φ*_0_. Here, we employ the same laser and plasma parameters shown in Fig. 2, except for the different initial phases of the signal laser pulse, which vary from 0 to π radians. Based on the CEP-stabilized laser pulses delivered by the kHz laser systems^43–46^, this scenario has the potential to produce multi-mJ few-cycle mid-IR pulses not only at relativistic intensities but also with stable CEPs and high repetition rates using our scheme, which are extremely beneficial for a wide range of applications.

The effect of the signal laser intensity on the infrared pulse generation is presented in Fig. 4b. It is worth noting that a non-relativistic signal laser pulse *a*_s0_ = 0.85 can also produce a relativistic mid-IR pulse *a*_ir_ ≈ 1.1 (with *λ*_ir_/*λ*_0_ ~ 4.1 and *E*_z,ir_/*E*_z0_ ~ 0.33), where ~34% of the signal laser energy is transferred into the output infrared pulse. With the increase in the signal laser intensity, the wavelength of the output pulse increases since the density gradient at the front of the second bubble becomes sharper. With the increase in the radiated infrared wavelength, the pulse normalized amplitude is significantly enhanced since *a*_ir_ ∝ *λ*_ir_*E*_z,ir_. This enhancement is an advantage of long-wavelength infrared pulses, the result of which is that the final output mid-IR pulse can easily have a higher normalized amplitude than that of the initial signal pulse despite the fact that the pulse is damped considerably. However, the initial signal laser intensity cannot be increased to an arbitrarily high value; otherwise, it will cause strong nonlinear coupling between the laser and plasma, thereby resulting in a large fraction of laser energy absorption and pulse attenuation. For example, when *a*_s0_ = 2.5, only ~26% (15 mJ) of the signal laser energy is converted into the infrared pulse, which is approximately 10% of the total energy of the signal pulse plus the drive pulse. Therefore, the incident signal pulse intensity should not be too high (i.e., $a_{s0}\, \lesssim\, 2.5$) for high-efficiency mid-IR pulse generation.

**Fig. 5:**
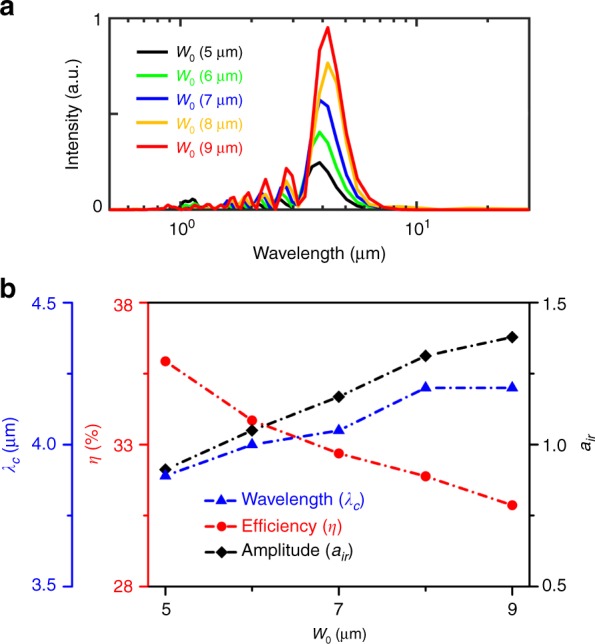
Effects of the spot size of the input signal pulse on the output mid-IR pulses. **a** The spectra of the output infrared pulses under different spot radii of the signal pulse. **b** The central wavelength (*λ*_c_), normalized amplitude (*a*_ir_), and energy conversion efficiency (*η*) of the mid-IR pulses as a function of the signal laser spot radius

Figure 5 illustrates the effect of the spot size of the signal laser pulse on the spectrum of the mid-IR pulses, where the spot radius (*w*_0_) is varied in the range from 5 to 9 μm, while all other parameters are the same as those presented in Fig. 2. It is shown that the spectral range of the produced mid-IR pulses is similar under different spot radii of the signal pulse, with a central wavelength of approximately 4 μm. This similarity means that the wavelength of the mid-IR pulse depends weakly on the laser transverse dimensions when the initial spot radius of the signal pulse is larger than the central wavelength of the resulting mid-IR pulses. The spectral intensity of the output mid-IR pulses increases with the spot size mainly due to the increase in the initial signal pulse energy, which also gives rise to an enhancement of the plasma wake excitation and thus causes a slight change in the frequency spectrum. It is noted that the spot size of the signal pulse should not be too large; otherwise, the part of the laser pulse outside the plasma bubble undergoes little frequency-downshifting and is depleted in the plasmas, which leads to a decrease in the energy conversion efficiency.

## Discussion

**Fig. 6:**
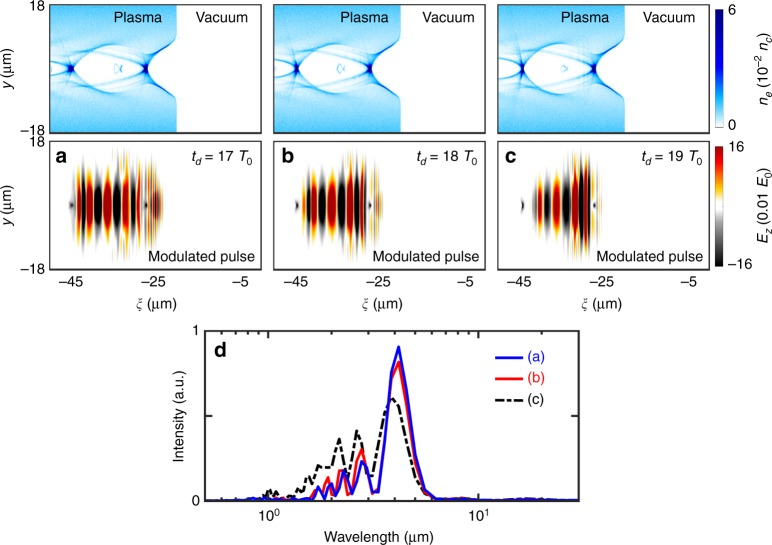
Effects of the initial pulse duration and time delay on the modulated pulse. The electron density (*n*_e_) in the upper row and the transverse electric field of the signal laser (*E*_z_) in the lower row are shown for three different delay times *t*_d_ = 17*T*_0_ (**a**), 18*T*_0_ (**b**) and 19*T*_0_ (**c**). **d** The spectra of the modulated signal pulses

To further demonstrate the robustness and feasibility of the proposed concept, we consider the same pulse duration of 10 fs (FWHM) for both the drive and signal pulses, and the corresponding pulse energy and peak power are respectively 55 mJ and 5.5 TW for the drive laser pulse and 13.7 mJ and 1.37 TW for the signal laser pulse. These laser pulses are readily achieved in experiments by using a dielectric mirror to split the main laser beam into a driver beam and the other signal beam with a certain time delay, which can be delivered by a multi-TW few-cycle laser system at a kHz repetition rate at existing laser facilities^44^. The time delay between the driver and signal beams can be precisely adjusted by using an optical delay stage. Here, we consider two pulses separated with three different delay times *t*_d_ = 17*T*_0_, 18*T*_0_ and 19*T*_0_, while all other parameters are the same as those in Fig. 2. It is interesting to see that there is no significant impact on the frequency-downshifting mechanism, and the resulting infrared pulses all reach a comparable level with spectral peaks at a wavelength of ~4 μm, as illustrated in Fig. 6. In the case of *t*_d_ = 17*T*_0_, one can see that there is also a distinct high-frequency part in the final modulated pulse. This exists because the front of the signal pulse has already been entered into the density down-ramp region with ∂[*n*_e_(*ξ*, *τ*)]/∂*ξ* < 0 at the end of the first bubble. With the increase in the delay time, the peak of the initial signal pulse will be away from the front toward the center of the second bubble, encountering a gentler density gradient. Therefore, it leads to a more moderate photon frequency downconversion. Once the initial signal laser directly resides in the bubble center (near the electron-free region), it is unlikely that its frequency will be modulated by much. Thus, the delay time should not be too long.

It is noted that the generated mid-IR pulses can be further collimated and focused by an off-axis parabolic mirror and separated by a germanium filter where the near-IR pulses are reflected. This may offer a way to deliver and/or measure the output mid-IR pulses for many applications. As a whole, these findings indicate that our scheme is robust and practical in producing relativistic few-cycle mid-IR pulses and makes it feasible to apply it to high-repetition-rate compact laser systems.

In addition, we also carried out some additional 3D PIC simulations to study the effects of the laser polarization direction and the plasma density up- and down-ramps on the mid-IR pulse generation. The details are illustrated in the Supplementary Information. We find that the photon frequency downconversion of the signal laser in a plasma optical modulator is insensitive to the laser polarization direction and plasma density ramps within a certain range (see Figs. S2, S3).

In conclusion, we have reported and numerically demonstrated a scheme to generate relativistic multi-mJ near-single-cycle mid-IR pulses based on plasma-based optical modulators using TW-class laser pulses. The resulting mid-IR pulses can be flexibly tuned by changing the initial laser and/or plasma parameters, and the energy conversion efficiency from the signal pulse to the mid-IR pulse can be as high as 30%. The advent of these intense mid-IR pulses has significant implications for light-matter interactions, moving them into a unique regime of long wavelength, few-cycle duration and relativistic intensity. As TW-class laser pulses can be delivered at a kHz repetition rate, they provide the ability to operate at a high repetition rate and thus enhance their availability and stability. This situation offers a unique opportunity for a variety of ultrafast and high field applications.

## Methods

### PIC simulations

The full 3D simulations were carried out with the electromagnetic relativistic PIC code EPOCH^54^, allowing the self-consistent simulation of laser-plasma interactions. The size of the simulation box is 50 μm(*x*) × 36 μm(*y*) × 36 μm(*z*) with grid cells of 1750 × 216 × 216 and four macro-particles per cell. The drive laser pulse is linearly polarized along the y-direction and has a spatial-temporal profile of ${\boldsymbol{a}}_{\mathrm{d}} = a_{{\mathrm{d}}0}{\mathrm{exp}}\left( { - \frac{{r^2}}{{w_0^2}}} \right){\mathrm{sin}}^2(\pi t/\tau _{{\mathrm{d}}0}){\mathbf{e}}_{\mathrm{y}}$ with a duration *τ*_d0_ = 10*T*_0_, spot size *w*_0_ = 8 μm, and normalized amplitude $a_{{\mathrm{d}}0} = \frac{{e\lambda _0E_{\mathrm{y}}}}{{2\pi m_{\mathrm{e}}c^2}} = 2$. These correspond to a peak intensity of 5.5 × 10^18^ W/cm^2^, peak power of 5.5 TW and pulse energy of 91.9 mJ at the laser wavelength *λ*_0_ = *cT*_0_ = 1 μm. The signal laser pulse is linearly polarized along the z-direction and has a similar profile of ${\boldsymbol{a}}_{\mathrm{s}} = a_{s0}{\mathrm{exp}}\left( { - \frac{{r^2}}{{w_0^2}}} \right){\mathrm{sin}}^2(\pi t/\tau _{{\mathrm{s}}0}){\mathbf{e}}_{\mathrm{z}}$ with $a_{{\mathrm{s}}0} = \frac{{e\lambda _0E_{\mathrm{z}}}}{{2\pi m_{\mathrm{e}}c^2}} = 1$, *τ*_s0_ = 4*T*_0_, and the same initial spot size and wavelength as those of the drive laser. These correspond to a signal pulse energy of 9.2 mJ and a peak power of 1.37 TW. The signal pulse is delayed by 21*T*_0_ from the drive pulse in this example. One notes that these laser pulses are readily available on the existing TW-class kHz-level laser systems^43–46^. To guide the propagation of the above focused laser pulses over many Rayleigh lengths, a plasma channel is adopted. The initial density profile of the plasma channel is given by $n_{\mathrm{e}} = n_0 + {\mathrm{\Delta }}n_0$ with a length of *L*_0_ = 1597 μm, where *n*_0_ = 3.5 × 10^−3^*n*_c_ is the background density, ${\mathrm{\Delta }}n_0 = \frac{{\lambda _0^2}}{{\pi ^2w_0^4}}r^2n_{\mathrm{c}}$ is the channel depth, and *r* is the radial distance from the channel axis. This type of plasma channel can be generated in several ways and has been widely used in laser-plasma experiments^55,56^.

## Supplementary information

Supplementary Informantion

## Data Availability

The data that support the findings of this study are available from the corresponding author upon request.
