# Supplementary material for: Efficient generation of relativistic near-single-cycle mid-infrared pulses in plasmas
Source: Light Sci Appl. 2020 Mar 20;9:46. doi: 10.1038/s41377-020-0282-3 (PMC7083853; doi:10.1038/s41377-020-0282-3)
Supplement: Supplementary file 1 — Supplementary Informantion [file 41377_2020_282_MOESM1_ESM.docx]

**Supplementary Informantion:**

**Efficient generation of relativistic near-single-cycle mid-infrared pulses in plasmas**

**Authors:**

Xing-Long Zhu^1, 2, 3^, Su-Ming Weng^1, 2*^, Min Chen^1, 2^, Zheng-Ming Sheng^1, 2, 3, 4, 5*^, and Jie Zhang^1, 2, 6^

**Affiliations:**

^1^ Key Laboratory for Laser Plasmas (MOE), School of Physics and Astronomy, Shanghai Jiao Tong University, Shanghai 200240, China

^2^ Collaborative Innovation Center of IFSA, Shanghai Jiao Tong University, Shanghai 200240, China

^3^ SUPA, Department of Physics, University of Strathclyde, Glasgow G4 0NG, UK

^4^ Cockcroft Institute, Sci-Tech Daresbury, Cheshire WA4 4AD, UK

^5^ Tsung-Dao Lee Institute, Shanghai 200240, China

^6^ Institute of Physics, Chinese Academy of Sciences, Beijing 100190, China

^*^Corresponding author. e-mail: wengsuming@sjtu.edu.cn (S.M.W.); z.sheng@strath.ac.uk (Z.M.S.)

**The spectral evolution of the drive laser pulse**

Figure S1 shows the spectral evolution of the drive laser pulse propagating in plasmas. One can see clearly that the frequency downshift of the drive pulse is much smaller than that of the signal pulse (shown in Fig. 2d in the manuscript), where about 1% of the drive pulse energy is converted into the output mid-IR pulses in the spectral range of above 3 μm.


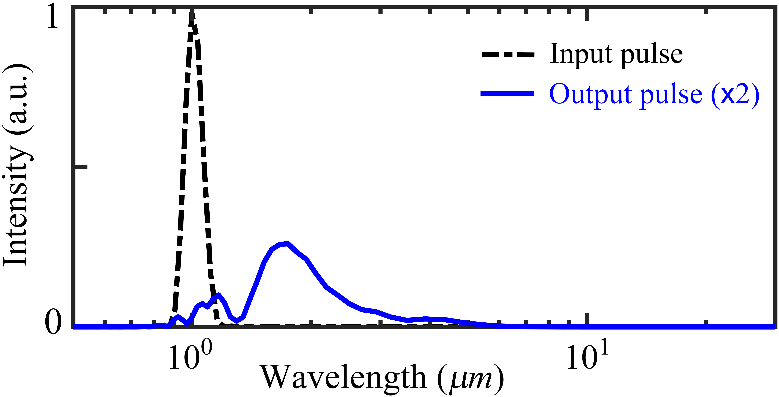


**Fig. S1. The spectral evolution of the drive laser pulse.** The initial and final spectra of the drive pulse propagating in plasmas. The spectral intensity of the output pule is multiplied by a factor of 2 (blue line).

**Effect of the laser polarization direction on the output mid-IR pulses**

We have carried out the 3D PIC simulations to study the effect of the laser polarization direction on the photon frequency downshifting in plasmas, where all the parameters are the same as those shown in Fig. 2 in the main text except for the polarization of the drive pulse and the signal pulse in the same direction. The results reveal that there is no considerable influence on the frequency downshift process, where the two cases of parallel and perpendicular polarizations for the two pulses produce comparable frequency shift and energy conversion efficiency with a certain CEP dependency relationship, as illustrated in Fig. S2 (for the parallel polarization case) and Fig. 2 (for the perpendicular polarization case), respectively.


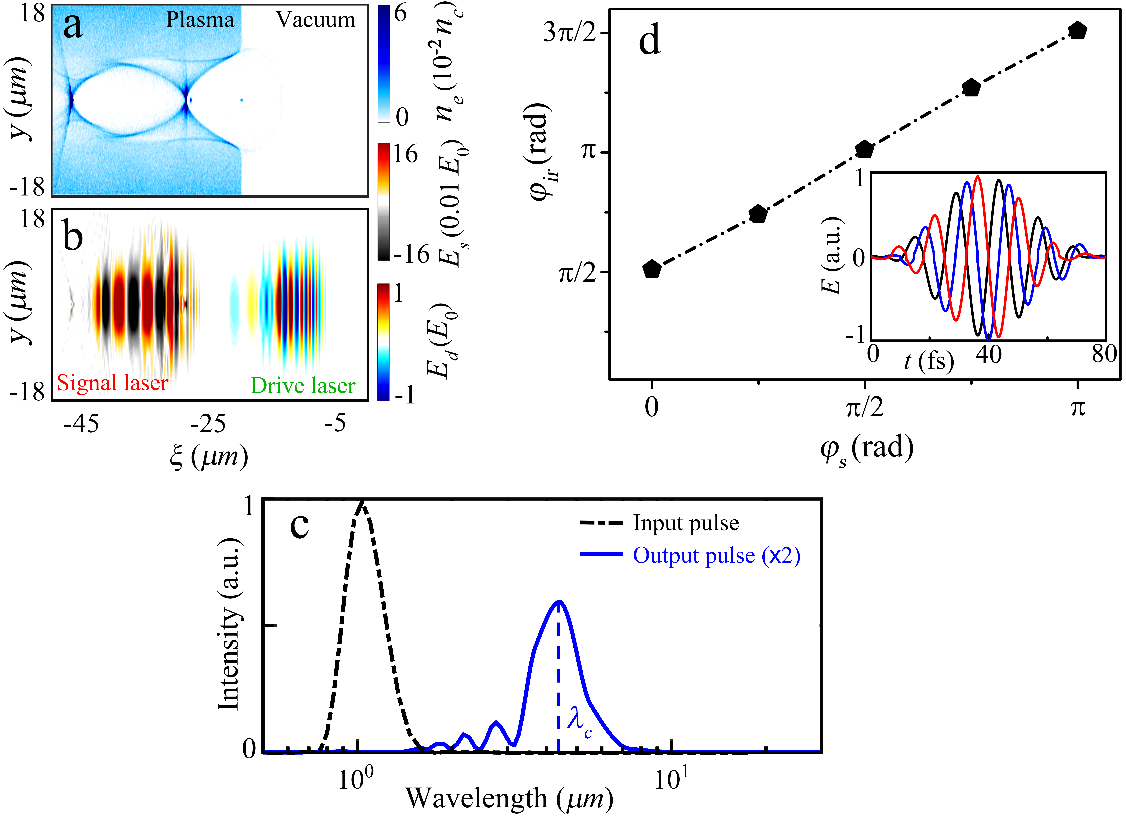


**Fig. S2. Effect of the laser polarization direction on the mid-IR pulse generation.** Distributions of the plasma density ($n_{e}$) in (**a**), and the transverse electric fields of the drive laser ($E_{d}$) and the signal laser ($E_{s}$) in (**b**). (**c**) The spectral evolution of the modulated signal pulse for the polarization of both the drive pulse and the signal pulse in the same direction. (**d**) Dependence of the CEP of the resulting mid-IR pulse on that of the initial signal pulse. The inset shows the electric field waveform of the mid-IR pulse at the central wavelength $\lambda_{c}\approx4.3 \mu m$ for different CEPs of the initial signal pulse (0, black line; $\pi/2$, blue line; $\pi$, red line).

**Effect of the plasma density ramps on the frequency downshift**

To consider the plasma edge effects, we have carried out an additional simulation by taking a longitudinal plasma density profile with a 50 μm up-ramp at the beginning, a 50 μm down-ramp at the end, and a 1550 μm plateau region, while all other parameters are unchanged, as illustrated in Fig. S3. One can see that there is no significant influence on the frequency down-conversion, where the output signal laser pulse has comparable spectral range and intensity as compared to the results presented in Fig. 2 in the main text.


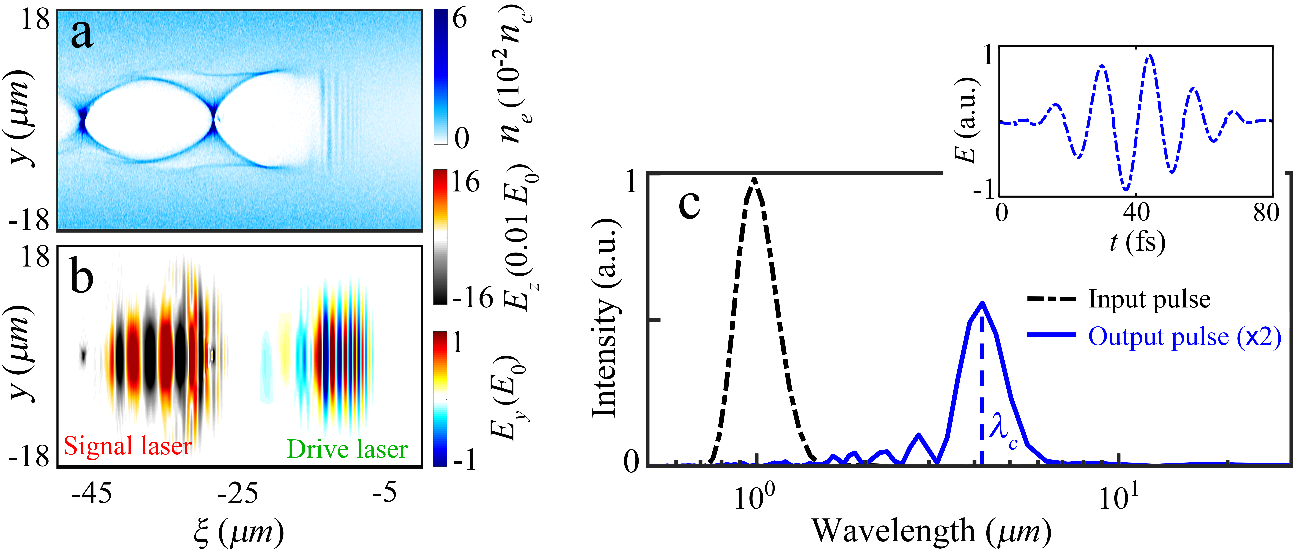


**Fig. S3. Effect of the plasma density ramps on the mid-IR pulse generation.** Distributions of the plasma density (**a**), and the transverse electric fields of the drive laser and the signal laser (**b**) at the position of $ct=1620 \mu m$. (**c**) Evolution of the initial (input pulse) and final (output pulse) spectra of the signal pulse modulated in the plasma wake. The inset shows the electric field waveform of the mid-IR pulse at the central wavelength $\lambda_{c}\approx4.2 \mu m$.
